# Supplementary material for: Site-Specific Antibody Conjugation to Engineered Double Cysteine Residues
Source: Pharmaceuticals (Basel). 2021 Jul 14;14(7):672. doi: 10.3390/ph14070672 (PMC8308878; doi:10.3390/ph14070672)
Supplement: Supplementary file 1 [file pharmaceuticals-14-00672-s001.zip › pharmaceuticals-1266460-supplementary.pdf]

Site-specific antibody conjugation to engineered double cysteine residues

(Supplementary files)

Qun Zhou[a], Josephine Kyazike[a], Ekaterina Boudanova[a], Michael Drzyzga[a], Denise Honey[a], Robert Cost[a], Lihui Hou[a], Francis Duffieux[b], Marie-Priscille Brun[c], Anna Park[a], Huawei Qiu[a]

[a]Large Molecules Research, Sanofi, Framingham, MA, USA; [b]Large Molecules Research, Sanofi, Vitry-Sur-Seine, France; [c]Integrated Drug Discovery, Sanofi, Vitry-Sur-Seine, France

Figure S1

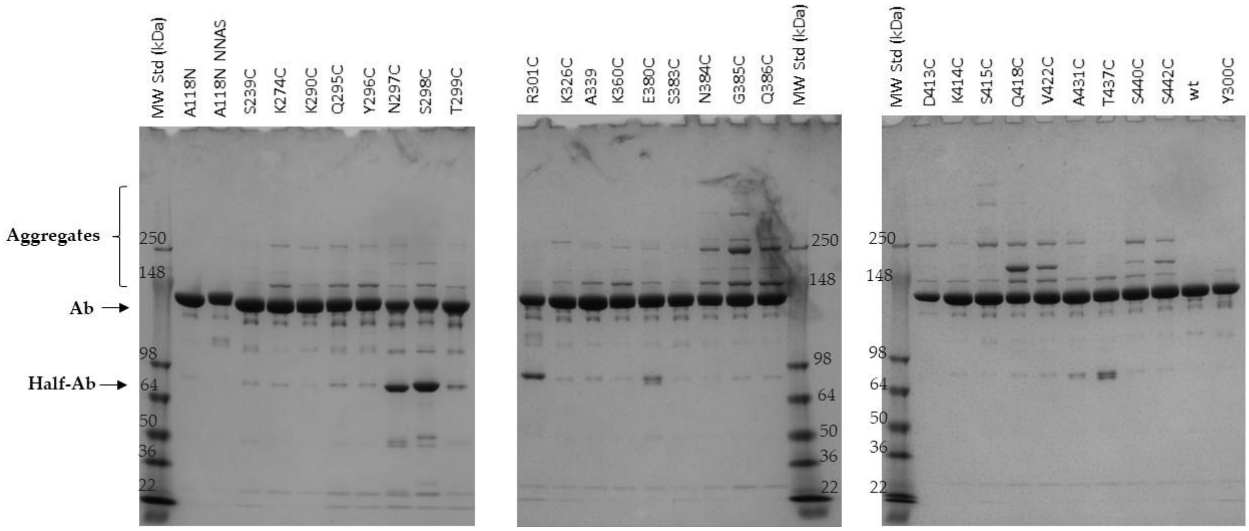

MW Std (kDa)
A118N
A118N NNAS
S239C
K274C
K290C
Q295C
Y296C
N297C
S298C
T299C
R301C
K326C
A339
K360C
E380C
S383C
N384C
G385C
Q386C
MW Std (kDa)
MW Std (kDa)
D413C
K414C
S415C
Q418C
V422C
A431C
T437C
S440C
S442C
wt
Y300C
Aggregates
Ab
Half-Ab
250
148
98
64
50
36
22

Figure S2

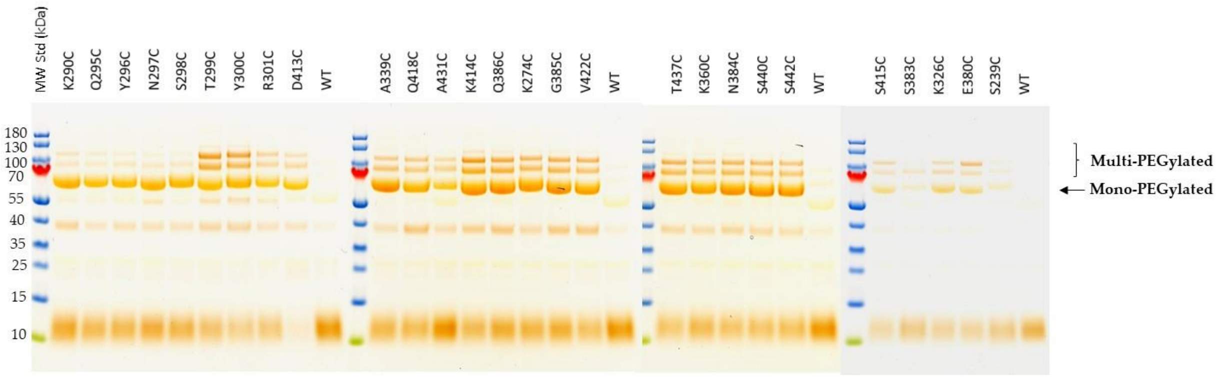
MW Std (kDa)
K290C
Q295C
Y296C
N297C
S298C
T299C
Y300C
R301C
D413C
WT
A339C
Q418C
A431C
K414C
Q386C
K274C
G385C
V422C
WT
T437C
K360C
N384C
S440C
S442C
WT
S415C
S383C
K326C
E380C
S239C
WT
180
130
100
70
55
40
35
25
15
10
Multi-PEGylated
Mono-PEGylated

Figure S3

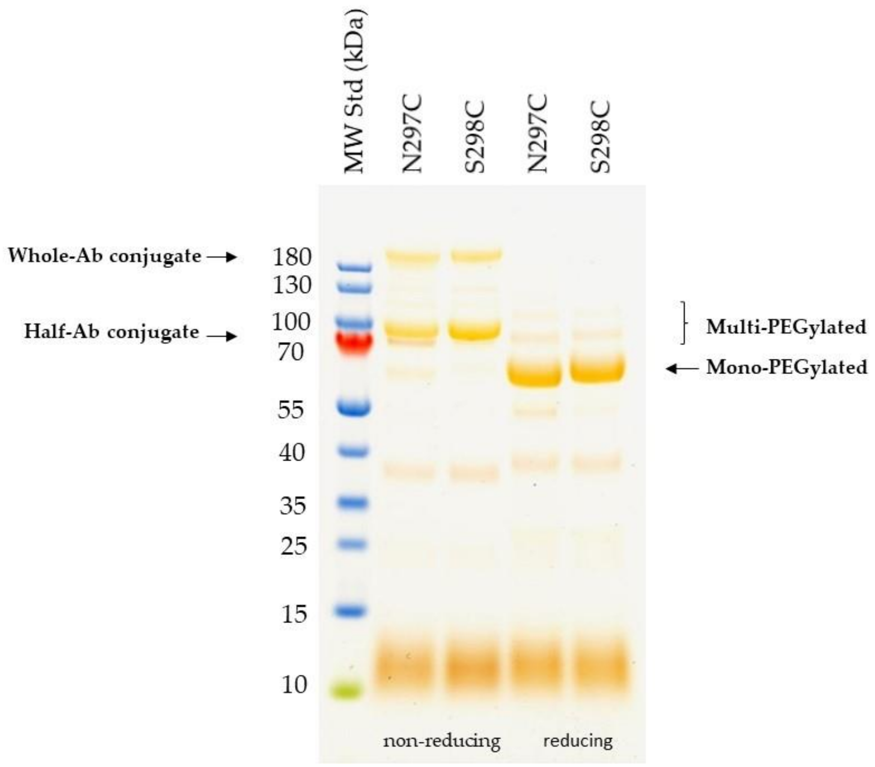

MW Std (kDa)
N297C
S298C
N297C
S298C
Whole-Ab conjugate
Half-Ab conjugate
180
130
100
70
55
40
35
25
15
10
Multi-PEGylated
Mono-PEGylated
non-reducing
reducing

Figure S4

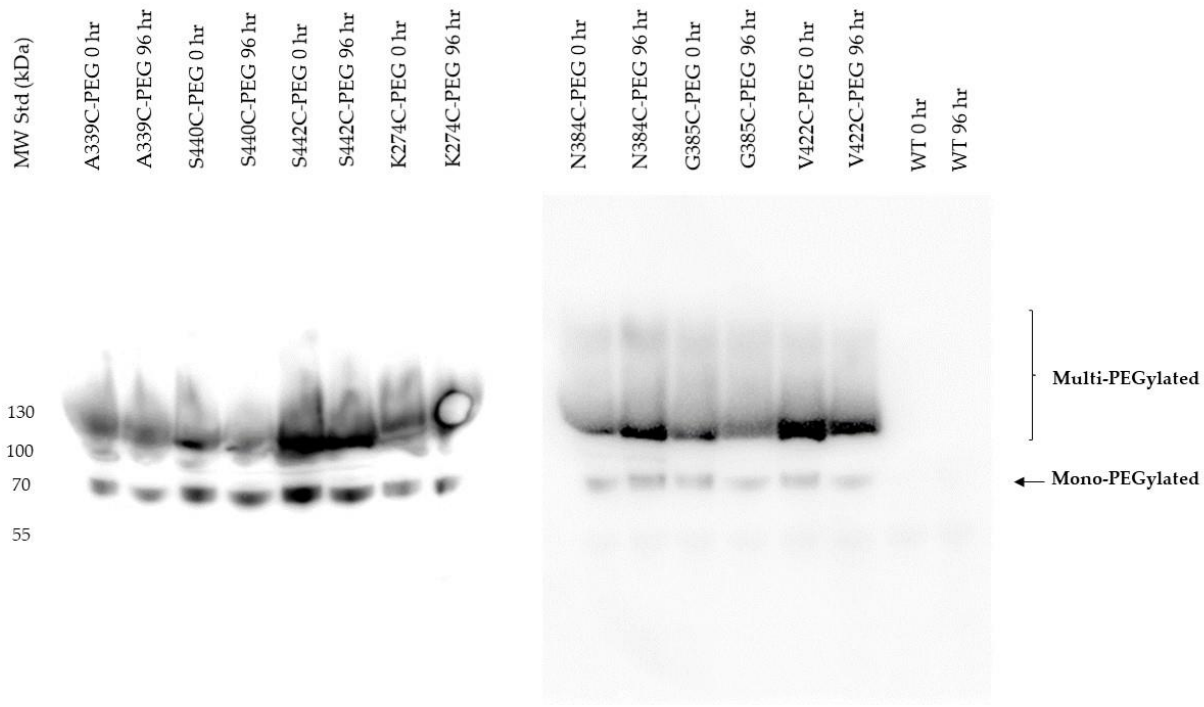
MW Std (kDa)
A339C-PEG 0 hr
A339C-PEG 96 hr
S440C-PEG 0 hr
S440C-PEG 96 hr
S442C-PEG 0 hr
S442C-PEG 96 hr
K274C-PEG 0 hr
K274C-PEG 96 hr
N384C-PEG 0 hr
N384C-PEG 96 hr
G385C-PEG 0 hr
G385C-PEG 96 hr
V422C-PEG 0 hr
V422C-PEG 96 hr
WT 0 hr
WT 96 hr
130
100
70
55
Multi-PEGylated
Mono-PEGylated

Figure S5

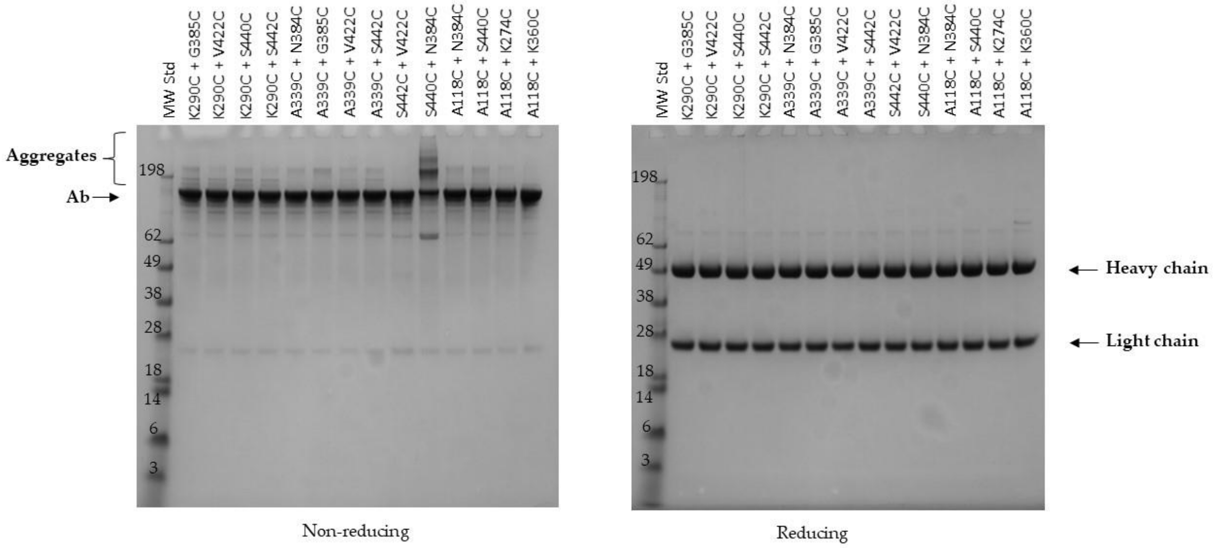
MW Std
K290C + G385C
K290C + V422C
K290C + S440C
K290C + S442C
A339C + N384C
A339C + G385C
A339C + V422C
A339C + S442C
S442C + V422C
S440C + N384C
A118C + N384C
A118C + S440C
A118C + K274C
A118C + K360C
Aggregates
Ab
198
62
49
38
28
18
14
6
3
Non-reducing
Reducing
Heavy chain
Light chain

Supplementary Figure Legends:

Figure S1: SDS-PAGE analysis of purified single cysteine mutants. 27 single cysteine mutants were purified using protein A and analyzed using 4-12% non-reducing Tris-Glycine SDS-PAGE. Wild-type antibody and hyperglycosylated mutants, A118N and NNAS, were used as controls. SeeBlue Plus 2 was used as protein molecular weight standards (MW Std) with different kDa as shown on the left of the gel.

Figure S2: SDS-PAGE and PEG staining of single cysteine mutant PEGylated. 27 single cysteine mutants PEGylated (13 μg each) were analyzed using 4-12% Bis-Tris NuPAGE under reducing conditions. The gels were stained using PEG staining as described in the Materials and Method. The wild-type antibody (WT), which was also PEGylated, was used as a control. PageRuler pre-stained protein ladder was used as protein molecular weight standards (MW Std) with different kDa as shown on left of the gel.

Figure S3: SDS-PAGE and PEG staining of two single cysteine mutants, N297C and S298C, after PEGylation. Two single cysteine mutants, N297C and S298C, were PEGylated and analyzed using 4-12% Bis-Tris NuPAGE under non-reducing and reducing conditions. The gel was stained using PEG staining as described in the Materials and Method. Significant amounts of half antibody conjugates were detected in the gel under non-reducing condition. PageRuler pre-stained protein ladder was used as protein molecular weight standards (MW Std) with different kDa as shown on left of the gel.

Figure S4: Western blot of PEGylated single cysteine mutants incubated with plasma. The samples (0.1 μg) from PEGylated antibodies and wild-type antibody incubated with mouse plasma were applied to SDS-PAGE under reducing condition and were analyzed using western

blot with anti-PEG antibody. The mono-PEGylated antibody bands, as shown by arrow, were analyzed using AlphaView software to determine the amount of PEG remained after incubation in plasma for 96 hours. The diffuse bands above the mono-PEGylated antibody bands are multi-PEGylated species which reacts strongly to the anti-PEG antibody due to the presence of additional PEG.

Figure S5: SDS-PAGE analysis of purified double cysteine mutants. The double cysteine mutants were analyzed using 4-12% Tris-Glycine SDS-PAGE under non-reducing and reducing conditions. SeeBlue Plus 2 was used as protein molecular weight standards (MW Std) with different kDa as shown on left of the gels.
